# Identification of Odor Active Compounds in *Physalis peruviana* L.

**DOI:** 10.3390/molecules25020245

**Published:** 2020-01-07

**Authors:** Małgorzata A. Majcher, Magdalena Scheibe, Henryk H. Jeleń

**Affiliations:** 1 Faculty of Food Science and Nutrition, Poznań University of Life Sciences, Wojska Polskiego 31, 60-624 Poznań, Poland; majcherm@up.poznan.pl; 2 Department of Analytical Chemistry, Chemical Faculty, Gdańsk University of Technology, Gabriela Narutowicza 11/12, 80-233 Gdańsk, Poland; magdalena.scheibe@gmail.com

**Keywords:** *Physalis peruviana* L., cape gooseberry, goldenberry, flavor, odor-active, aroma, sensory analysis, SAFE, HS-SPME, GC-O, GC-MS

## Abstract

The volatiles of cape gooseberry fruit (*Physalis peruviana* L.) were isolated by solvent-assisted flavor evaporation (SAFE), odor active compounds identified by gas chromatography–olfactometry (GC-O) and gas chromatography–mass spectrometry (GC-MS). Quantitation of compounds was performed by headspace—solid phase microextraction (HS-SPME) for all but one. Aroma extract dilution analysis (AEDA) revealed 18 odor active regions, with the highest flavor dilution values (FD = 512) noted for ethyl butanoate and 4-hydroxy-2,5-dimethylfuran-3-one (furaneol). Odor activity values were determined for all 18 compounds and the highest was noted for ethyl butanoate (OAV = 504), followed by linalool, (*E*)-non-2-enal, (*2E,6Z*)-nona-2,6-dienal, hexanal, ethyl octanoate, ethyl hexanoate, butane-2,3-dione, and 2-methylpropanal. The main groups of odor active compounds in *Physalis peruviana* L. were esters and aldehydes. A recombinant experiment confirmed the identification and quantitative results.

## 1. Introduction

Fruits, especially berries, have been shown to provide important benefits because of their high content of vitamins, minerals, and antioxidants [1]. The fruits featured by the highest content of nutrient-rich compounds are commonly known as the “superfruits”, and supplementation of the human diet with superfruits provides many health benefits [2]. In well-developed countries, superfruits is more of a marketing rather than a scientific term and promotes the demand for rare fruits that can be consumed and used as ingredients by manufacturers of functional foods, nutraceuticals, and beverages. Therefore, food and medicinal products based on these kinds of fruit are growing more popular among consumers. They can be used to enrich the diet with new flavors while providing many natural ingredients which exhibit significant health benefits [2].

Polyphenols with a high antioxidant capacity are the most common compounds, which can be found in superfruits. Apart from the aforementioned compounds, superfruits contain a significant content of different terpenes, which determine the flavor of fruits and exhibit bioactive properties. Among others, α-phellandrene and β-myrcene exhibit antioxidant properties, limonene and *p*-cymene have antimicrobial, antidiabetic, antifungal, antibacterial, anti-nociceptive, and anti-inflammatory properties, respectively [3,4,5,6]. Considerable interest led to the increase of the number of research and publications focusing on the health benefits of superfruits [7,8].

Currently, one of the most interesting is *Physalis peruviana* L., which is a solanaceous, herbaceous, semi-shrub, upright, and perennial in subtropical zones plant, native to tropical South America. In Colombia, *P. peruviana* L.(known as a cape gooseberry or goldenberry) is cultivated in regions between 1500 m and 3000 m above sea level and can grows until it reaches 0.6 m to 0.9 m, and in some cases can grow up to 1.8 m. Cape gooseberry fruits are round berries with an average diameter of 20–25 mm and an approximate weight of 4–5 g, protected by an accrescent calyx and covered by a brilliant resinous-yellow peel, containing inside around 100 to 200 small seeds [9,10]. Fruits possess antispasmodic, diuretic, antiseptic, sedative, analgesic properties, and also help to fortify the optic nerve, throat trouble relief, and elimination of intestinal parasites and amoeba. There have also been reported antidiabetic properties, recommending the consumption of five fruits a day. There are studies indicating that eating the fruit of the cape gooseberry reduces blood glucose after 90 min postprandial in young adults, causing a greater hypoglycemic effect after this period [11]. The cape gooseberry is extensively used as a medicinal herb for treating diseases such as cancer, malaria, asthma, hepatitis, dermatitis, and rheumatism [12]. So far, there are no studies that indicate possible adverse effects. Generally, *P. peruviana* L. is an attractive fruit for international markets thanks to its important nutritional and medicinal properties. Nowadays, the berries can be found in the form of jams, raisins, and chocolate-covered candies, juices, pomace, and other products, or sweetened with sugar as a snack [13].

In our previous research, we carried out a study on the terpene profile in *P. peruviana* L. berries, extracted and estimated via different preparation and analytic methods [14,15]. This work is focused on the identification of odor-active flavor compounds in cape gooseberries using the molecular sensory approach [16]. The volatile fraction of fruit was obtained by applying solvent-assisted flavor evaporation (SAFE) and headspace solid-phase microextraction (HS-SPME) techniques [16,17], and analyzed via gas chromatography-olfactometry (GC-O) with aroma extract dilution analysis (AEDA) and gas chromatography-mass spectrometry (GC-MS) methods [18,19]. Odor activity values (OAV) for the key odorants were determined.

## 2. Results

### 2.1. Sensory Characterization of Cape Gooseberry Fruit (Physalis peruviana L.)

**Figure 1:**
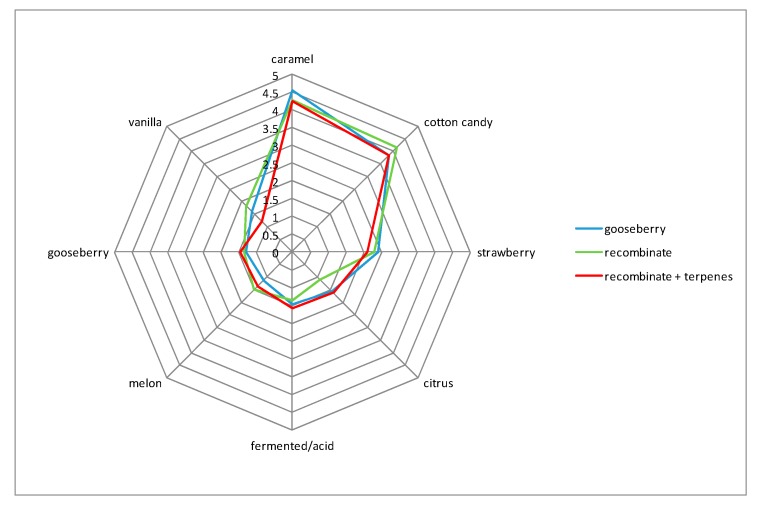
Sensory aroma profiles of a cape gooseberry fruit, its recombinant, and recombinant with addition of terpenes.

In the first experiment, the cape gooseberry fruit was subjected to aroma profile analysis. For that reason, eight odor descriptors, caramel, cotton candy, strawberry, citrus, fermented/acid, melon, gooseberry (associated with *Ribes uva-crispa* fruit), and vanilla, chosen in a preliminary section, were evaluated by the sensory panel. Results presented in Figure 1 illustrate that the overall aroma of the *P. peruviana* fruit pronounced strong caramel and cotton candy notes followed by strawberry-like, citrus, acid, and vanilla notes.

### 2.2. Screening the Cape Gooseberry Volatiles for Aroma-Active Compounds by the Means of GC-O AEDA Analysis

Aroma isolates obtained using SAFE extraction were submitted to GC-O and AEDA analysis, which revealed 18 aroma-active compounds. The authenticity of them was confirmed by GC-O and GC-MS analysis on two columns of different polarity (SPB-5 and Supelcowax-10) and the comparison of retention indices (RI), mass spectra (MS), and odor with authentic standards (Table 1). However, in one case of 2-acetyl-1-pyrroline, the MS signal in full scan was too weak to obtain a good quality spectrum, so it was identified based on all aforementioned methods with the exception of MS spectra comparison. Flavor dilution factors (FD) for analyzed compounds were obtained by sniffing the serially diluted SAFE extracts, which ranged from 4 to 512. Among odorants, two compounds exhibited the highest FD factor (512)—cotton candy smelling furaneol and fruity smelling ethyl butanoate. Linalool was the only odorant representing terpenoids with a FD of 256. FD values of 128 were attributed to ethyl octanoate, 3-methylsulfanylpropanal (methional), and 2,3-diethyl-5-methylpyrazine.

**Figure 2:**
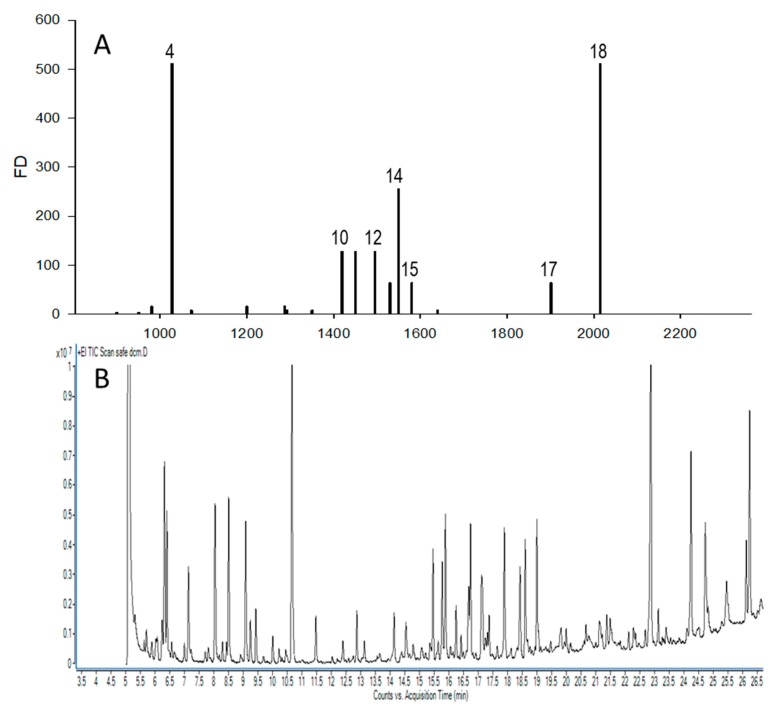
Compilation of aromagram (**A**) and TIC chromatogram (**B**) of odor active and volatile compounds in cape gooseberry fruit. Compounds number on (**A**) correspond to those in Table 1.

Figure 2 shows the aromagram (A) with FD values and the total ion current (TIC) GC-MS chromatogram on Supelcowax-10 column (B) of SAFE extract. Aromagram represents intensities of compounds sniffed at olfactory port of GC-O with AEDA approach and shows the location of key odorants on the GC-MS chromatogram of volatile compounds.

To determine the odor activity values (OAV) for compounds identified using AEDA procedure, quantitation was performed using headspace solid phase microextraction (HS-SPME) with a standard addition method for all compounds but one (furaneol). To quantify compounds of interest, their linear range was determined. Standards of compounds of interest were added to sample to construct calibration curve with its intercept, indicating concentration of particular compounds. Concentration ranges of added standards were determined based on peak areas of compounds of interest. The highest linear ranges were noted for esters—ethyl butanoate and ethyl octanoate—up to 3000 and 2000 µg/kg, respectively (addition of 500–3000 and 100 to 2000 µg/kg concentrations, respectively). Hexanal and butane-2,3-dione were linear up to 1000 µg/kg, whereas for the majority of the remaining compounds, a linear range of up to 200 µg/kg was achieved. For oct-1-ene-3-ol, ethyl 2-methylpropanoate linearity ranged up to 20 µg/kg. For ethyl butanoate and ethyl hexanoate the regression coefficient of the curve was 1.000, for hexanal, ethyl octanoate, and 2-phenylacetaldehyde was 0.999, and only for octanal (r^2^ = 0.969), (*E*)-non-2-enal (0.968), and 2-phenylethanol (0.978) were lower than 0.99. As it was impossible to get a reliable standard addition curve for 2,3-diethyl-5-methyl pyrazine its peak area (HS-SPME) was compared to 0.1 µg/kg of authentic standard. It was assumed that its concentration was <0.1 µg/kg, as 2,3-diethyl-5-methyl pyrazine peak in sample had a smaller area than the standard. Similarly, the concentration of 2-acetyl-1-pyrroline was assumed to be less than 0.01 µg/kg, as it was not possible to observe 2-acetyl-1-pyrroline significant peak in the fruit sample. Furaneol was the only compound quantified with a SIDA approach using deuterated analogue ^13^C_2_-furaneol.

Based on quantitative analysis and OT (in water, [20]), OAV values for 15 compounds >1 were calculated (Table 2). The highest OAV value was noted for ethyl butanoate, followed by linalool. High OAV values were also noted for aldehydes—(*E*)-non-2-enal, (*E*,*Z*)-nona-2,6-dienal, and hexanal. To verify the OAV results, a reconstitution experiment was prepared. As can be seen on Figure 1, the odor notes and their intensities for gooseberry and the recombinant closely resembled each other. Also, an additional sample was prepared using recombinant, to which terpenes identified earlier in gooseberry [14] were added. In this case, no significant differences were noted, proving that none of the numerous terpenes identified earlier contributed significantly to the flavor of cape gooseberry.

## 3. Discussion

Experiments devoted to analysis of aroma/volatile compounds of cape gooseberry were carried out by several authors. However, in gooseberry aroma analysis, no complete approach consisting of AEDA and OAV has been performed. Berger and colleagues [21] analyzed volatile compounds present in cape gooseberry, and also those responsible for the aroma already in 1989, employing a complex extraction procedure for the isolation of compounds of interest. It allowed authors to identify 5 terpene hydrocarbons, 28 alcohols (including 6 terpene ones), 12 aldehydes, 13 ketones, 26 acids, 51 esters, 7 lactones, and 5 other compounds. Interestingly, among volatiles identified, 8–12 odor impressions were recognized and compounds responsible for them were identified. Methyl-2-methylbutanoate (fruity), methoxy furanone (caramel), 3-methylbutyric acid (sweaty), β-damascenone (raspberry-like), β-ionone (violet), 4-octanolide (coconut), 5-octanolide (sweet-nutty), and hydroxyfuranone (roasted almonds) were the main odoriferous compounds described by authors, however their mutual concentrations/intensities have not been determined. The complex extraction procedure resulted in identification of odor active lactones, 3-methylbutyric acid, as well as damascenone and β-ionone, which were not identified in our study. This could be a result of either sample preparation differences and/or other composition of gooseberry fruit having been analyzed.

Liquid/liquid extraction using dichloromethane was also used for extraction of volatile and flavor compounds by Ylmaztekin [22], who identified 83 compounds in fruit of *Physalis peruviana*, where dominating (in area%) were alcohols and lactones, followed by esters and terpenes. Ylmaztekin also investigated main odorants, determining OAV values for them. Gamma octalactone (4-octanolide) was described as fruity, apricot had the highest OAV of 46.9, followed by gamma hexalactone (4-hexanolide), which was described as creamy, coconut (OAV 6.1). The next important odorants identified were ethyl octanoate, 2-heptanone, nonanal, hexanal, and citronellol (all with OAV >2), followed by 2-methyl-1-butanol, benzyl alcohol, 2-phenylethanol, 1-heptanol, ethyl decanoate, and 1-butanol (with OAV 1.0–1.6). Hexanal, ethyl octanoate, and 2-phenylethanol were identified in our study as key odorants as well. When the extraction method was changed by the same author from LLE to SPME^28^, the area % of particular groups of compounds substantially changed as well: the amount of lactones decreased tenfold (from 24.06 to 2.09%), amount of acids decreased from 5.05 to 0.90%, whereas the area % of esters substantially increased (from 11.7 for LLE to 38.5 for SPME). Similarly, the area% of aldehydes and ketones increased when SPME was used for extraction [23].

When volatile compounds of cape gooseberry were investigated using other sorbent-based techniques, in tube extraction (ITEX) terpenes prevailed among the main volatiles [14]. Out of 24 compounds which were quantified using calibration curves, 14 were terpene hydrocarbons or alcohols, followed by 4 esters, 4 alcohols, and 2 aldehydes. Interestingly, the most abundant compound in the examined cape gooseberry was benzaldehyde, followed by ethyl butanoate, 2-methyl-1-butanol, and 1-hexanol.

Our results, which are the first using AEDA to select odor active compounds, show differences in the compounds assumed as key odorants in cape gooseberry compared to previous works. The most striking difference was the absence of lactones in fruits examined by us. The differences noted can be attributed to various extraction methods applied in presented studies, as well as to differences in composition of particular fruit cultivars originating from Colombia, Turkey, or other countries or artefacts formation (as in the case of lactones). The reconstitution experiment indicated no significant differences between fruit aroma and the one obtained from reconstituted aroma based on compounds identified. The study broadens our knowledge on the flavor of exotic fruits flavor and indicates the importance of ethyl butanoate β-linalool, (*E*)-non-2-enal, (2*E*, 6*Z*)-nona-2,6-dienal, and hexanal as the compounds with the highest OAV values in gooseberry aroma creation, which may serve as sensory quality markers for this fruit.

## 4. Materials and Methods

### 4.1. Fruit Samples

Samples of cape gooseberry (*Physalis peruviana* L.) imported from Colombia and purchased at a local supermarket were analyzed. Before analysis, fruit samples (calyx removed) were stored in the freezer at −25 °C.

### 4.2. Chemical Standards

Solvents, such as methylene chloride and sodium sulfate were obtained from Sigma Aldrich (Poznań, Poland). A homologous series of C6-C24 n-alkanes and the following reference aroma compounds were purchased from Sigma-Aldrich (Poznań, Poland): 2-methylpropanal, ethyl 2-methyl propanoate butane-2,3-dione, ethyl butanoate, hexanal, ethyl hexanoate, octanal, oct-1-en-3-ol, ethyl octanoate, 3-methysulfanylpropanal (methional), 2,3-diethyl-5-methylpyrazine, (*E*)-non-2-enal, 3,7-dimethylocta-1,6-dien-3-ol (β-linalol), (*2E,6Z*)-nona-2,6-dienal, 2-phenylacetaldehyde, 2-phenylethanol, 4-hydroxy-2,5-dimethyl-3-furanone (furaneol), terpinen-4-ol, α-terpineol, β-myrcene, α-terpinolene, β-pinene, limonene, geraniol, β-citronellol, and γ-terpinene. Standards of 1-(3,4-dihydro-2*H*-pyrrol-5-yl)ethanone (2-acetyl-1-pyrroline) and isotopically labelled ^13^C_2_-furaneol were purchased from Aroma LAB (Freising, Germany).

### 4.3. Isolation Method

For isolation of volatile/odor active compounds, two methods were used: solvent assisted flavor evaporation (SAFE) and solid phase microextraction (SPME) [16,19]. Prior to SAFE, fruit samples (50 g) were frozen in liquid nitrogen (to quench enzymatic reaction), ground in a mortar and pestle into fine powder, and extracted with methylene chloride (300 mL) for 2 h each by shaking it in a horizontal shaker. Afterwards, volatiles were isolated by SAFE distillation and extract was dehumidified with an anhydrous sodium sulfate and concentrated with a Kuderna Danish concentrator (Sigma-Aldrich) to about 400 μL. The extract flavor resembled that of the fruit. For SPME analysis, 10 g of prepared fruit powder sample was placed in a 20 mL headspace vials and capped with PTFE/silicon septa caps. Extraction of volatiles was performed with CAR/PDMS/DVB fiber (Supelco) at 30 °C during 30 min using Combipal-type autosampler (Agilent Technologies, Willmington, DE, USA).

### 4.4. Gas Chromatography-Olfactometry (GC-O)

Odor active compounds were identified from SAFE extracts by GC-O on an HP 5890 gas chromatograph using the following capillary columns SPB-5 (30 m × 0.53 mm × 1.5 μm,) and Supelcowax-10 (30 m × 0.53 mm × 1 μm); both from Supelco, Bellefonte, PA, USA. The GC was equipped with an Y splitter dividing effluent between in-lab built olfactometry port with humidified air as a make-up flow (30 mL/min), and a flame ionization detector. The operating conditions were as follows for the SPB-5 column: initial oven temperature 40 °C (1 min), raised at 9 °C/min to 180 °C and at 20 °C/min to 280 °C. Operating conditions for the Supelcowax-10 column were as follows: initial oven temperature, 40 °C (2 min), raised to 240 °C at 9 °C/min rate, held for 2 min isothermally. For all peaks and flavor notes, retention indices were calculated to compare results obtained by GC/MS with literature data. Retention indices were calculated for each compound using a homologous series of C6–C24 n-alkanes.

### 4.5. Gas Chromatography/Mass Spectrometry

The chemical compounds were identified using GC/MS system (Agilent Technologies, Palo Alto, CA, USA): 7890A GC coupled to a 5975C MSD with a Supelcowax-10 column (30 m × 0.25 mm × 0.25 µm) or SLB-5MS (25 m × 0.2 mm × 0.33 µm) column (Supelco, Bellefonte, PA, USA). Operating conditions for GC/MS were as follows: He flow, 32.2 cm/sec; GC/MS interface, 260 °C, and 280 °C for polar and non-polar columns, respectively. Oven conditions were the same as for GC-O. Mass spectra were recorded in an electron impact mode (EI, 70 eV) in a scan range of *m*/*z* 33-350. For SPME fiber desorption, 260 °C temperature was used with splitless injection into 0.75 mm SPME liner. For all the volatiles except 2-acetyl-1-pyrroline, identification was performed by comparison of mass spectra, retention indices (RI), and odor notes on two columns of different polarities with standards. For 2-acetyl-1-pyrroline, the MS signal of the analyte in the sample was too weak to facilitate mass spectra comparison. In this case, only RI and odor notes of the compound were compared with authentic standard and used in its tentative identification.

### 4.6. Aroma Extract Dilution Analysis (AEDA)

The flavor dilution factor (FD) of each of the odorants was determined by AEDA^22^. The flavor extract (2 μL) was injected into a GC column. Odor-active regions were detected by GC-effluent sniffing (GC-O), and three panelists determined the description of the volatiles. The extract was than stepwise diluted (4, 8, 16, 32, etc.) by addition of methylene chloride, and each sample of the dilution series was analyzed until no odor was perceivable at the sniffing port. For each odor note, FD factor has been assigned, corresponding to the dilution factor of the highest diluted sample in which the odor was detectable.

### 4.7. Quantitation of Odorants

For all of the compounds except furaneol, 2-acetylpyrroline, and 2,3-diethyl-5-methylpyrazine, HS-SPME extraction has been performed with standard addition method (standard compounds in increasing concentrations were spiked into fruit sample to get calibration curve from which the concentration of particular compound can be obtained. Linearity for the standard curves was calculated as the regression coefficient (r^2^). Furaneol was quantified based on SAFE using added labelled standard of ^13^C_2_ furaneol. Quantitation was based on *m*/*z* 128 for furaneol and *m*/*z* 130 for its isotopologue.

### 4.8. Aroma Recombination

For cape gooseberry aroma recombination, stock solution of aroma compounds with OAV >1 was prepared in water with an adjusted level of sugar (11 g/100 g) and pH was adjusted to 3.9 with citric acid, and mixed in the concentration levels equal to those determined in the cape gooseberry sample.

### 4.9. Sensory Evaluation

Sensory analyses of cape gooseberry samples, as well as recombinants, were evaluated by 10 panelists with experience in sensory descriptive analysis. Aroma profile analyses were performed by orthonasally scoring eight odor qualities on a 10 cm linear scale anchored on either side for the intensity of attributes as “none” and “very strong”. The odor descriptors were determined in preliminary tests. The 5 g of (sliced) fruit sample or recombinant were placed in 100 mL glass containers and presented to the panelists, in a way that panelists could not see the content of the vessel. The results from the linear scale were converted into numerical values for data analysis.

## Figures and Tables

**Table 1:** Key odorants identified in cape gooseberry fruit using gas chromatography—olfactometry with aroma extract dilution analysis (GC-O AEDA).

No	Compound	odor	RI Supelcowax-10	RI SPB-5	FD
1	2-Methylpropanal	fruity	900	<600	4
2	Ethyl 2-methyl propanoate	fruity, anise-like	950	750	4
3	Butane-2,3-dione	buttery	980	<600	16
4	Ethyl butanoate	fruity	1027	802	512
5	Hexanal	fresh grass	1072	803	8
6	Ethyl hexanoate	fruity	1200	1002	16
7	Octanal	rancid, citrus	1287	998	16
8	Oct-1-en-3-ol	mushroom	1292	978	8
9	2-Acetyl-1-pyrroline	popcorn	1350	911	8
10	Ethyl octanoate	fruity	1419	1195	128
11	Methional	boiled potato	1450	907	128
12	2,3-Diethyl-5-methyl pyrazine	earthy	1495	1156	128
13	(*E*)-Non-2-enal	fatty, green	1530	1159	64
14	β-Linalool	fruity	1550	1101	256
15	(*E2, Z6*)-Nona-2,6-dienal	cucumber	1580	1150	64
16	2-Phenylacetaldehyde	rosy, honey-like	1640	1048	8
17	2-Phenylethanol	flowery, honey-like	1901	1118	64
18	Furaneol	cotton candy	2015	1080	512

RI—Retention indices on a Supelcowax-10 and SPB-5 columns.

**Table 2:** Concentration, odor thresholds, and odor activity values of aroma active compounds of cape gooseberry fruit.

Compound	OT ^a^ [µg/L]	Concentration ^b^ [µg/kg]	OAV ^c^
Ethyl butanoate	0.76	383.0	504
β-Linalol	0.089	32.0	360
(*E*)-Non-2-enal	0.08	24.0	300
(2*E, 6Z*)-Nona-2,6-dienal	0.02	5.0	250
Hexanal	4.50	450.0	100
Ethyl octanoate	5.00	411.0	82
Furaneol	30.0	1350.0	45
Ethyl hexanoate	1.20	45.0	38
Butane-2,3-dione	15.0	500.0	33
2-Methylpropanal	1.90	50.0	26
Octanal	0.70	15.0	21
Ethyl 2-methyl propanoate	0.089	1.8	20
Methional	0.20	36.0	18
2-Phenylacetaldehyde	4.00	25.0	6
Oct-1-en-3-ol	1.00	3.0	3
2,3-Diethyl-5-methyl pyrazine	1.00	<0.1	<1
2-Phenylethanol	1000	65.0	<1
2-Acetyl-1-pyrroline	0.1	<0.01	<1

^a^—odor thresholds in water [20]. ^b^—mean values based on three replicates with RSD value ≤12%. ^c^—odor activity values calculated by dividing the concentration of an analyte by its odor threshold value.
